# Targeted Metabolomic and Biochemical Changes During Nitrogen Stress Mediated Lipid Accumulation in *Scenedesmus quadricauda* CASA CC202

**DOI:** 10.3389/fbioe.2020.585632

**Published:** 2020-10-19

**Authors:** Sujitha Balakrishnan Sulochana, Muthu Arumugam

**Affiliations:** ^1^Microbial Processes and Technology Division, Council of Scientific and Industrial Research – National Institute for Interdisciplinary Science and Technology, Trivandrum, India; ^2^Academy of Scientific and Innovative Research, Ghaziabad, India

**Keywords:** microalgae, biofuel, nitrogen stress, Reactive Oxygen species, mitochondria, metabolic changes

## Abstract

*Scenedesmus quadricauda* CASA CC202, a potent freshwater microalga is being used as a biofuel feedstock, which accumulates 2.27 fold lipid during nitrogen stress induction. Upon nitrogen starvation, *S. quadricauda* undergoes biochemical and metabolic changes that perturb the cell to cope up the stress condition. The nitrogen stress-induced biochemical changes in mitochondrion exhibits due to the oxidative stress-induced Reactive Oxygen species (ROS) generation at high membrane potential (Δψ_m_). The predominant ROS generated during nitrogen starvation was H_2_O_2_, OH^–^, O_2_⋅^−^ and to suppress them, scavenging enzymes such as peroxidase and catalase increased to about 23.16 and 0.79 U/ml as compared to control (20.2, 0.19 U/ml). The targeted metabolic analysis showed, stress-related non-proteinogenic amino acids and energy equivalents elevated during the initial hours of nitrogen starvation. The nitrogen stress-triggered biochemical and metabolic changes along with other cellular events eventually lead to lipid accumulation in *S. quadricauda*.

## Graphical Abstract

Selective metabolomic and biochemical changes during nitrogen stress in *microalga*.

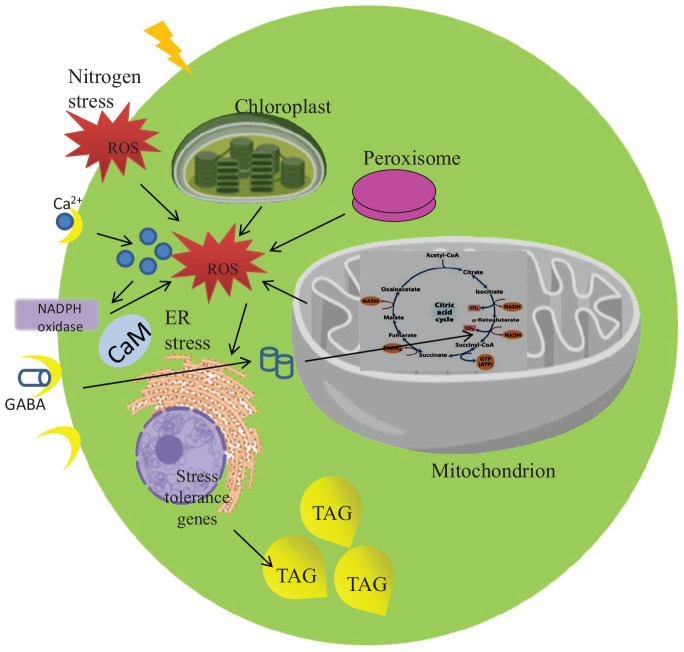

## Introduction

Microalgae are renowned as biofuel feedstock as it has the potential to meet current energy requirements. It has been well addressed that they accumulate increased lipid content per cell. The storage lipids in microalgae were synthesized in two steps such as *de novo* synthesis of fatty acids in plastids and triacylglycerol (TAG) biosynthesis in endoplasmic reticulum (ER). The Acetyl CoA from Calvin cycle is converted into Malonyl ACP and by the action of fatty acid synthase complex fatty acids were synthesized and free fatty acids were released in the plastid. Further the free fatty acids were entering into the ER and TAG synthesis by Kennedy pathway occurs (Wang et al., 2018). When an oleaginous (oil-producing) microalgae exposed to nitrogen stress which accumulates more lipid as an energy reserve (Lim et al., 2012). One such microalga, *Scenedesmus quadricauda* CASA CC202 which accumulates about 2.27 fold lipid during nitrogen starvation (Anand and Arumugam, 2015). In order to adapt the harsh environmental stimuli, several stress-responsive changes were occurring in the cell. Primarily the biomolecules such as lipid and carbohydrate level increase with a reduction in protein and photosynthetic pigments under nitrogen starvation (Msanne et al., 2012; Anand and Arumugam, 2015).

The actual biochemical and metabolic events in nitrogen stress mediated lipid accumulation and other abiotic stress is poorly addressed. It is complex cascade of reactions resulting in an increased lipid accumulation. An array of cellular events that switches on the lipid biosynthesis pathway to maintain the C/N homeostasis of the cell. The initial stress markers and signaling molecules lead to a tremendous rearrangement of metabolic pathways. The byproduct of these metabolic rearrangements is mainly activating the energy-saving shunt pathways and its associated reactions (Sweetlove et al., 2002; Bolton, 2009; Recht et al., 2014). Also, these stimulate an increased carbon channeling into fatty acid synthesis under nitrogen starvation. Therefore, a selective metabolomic study, is envisioned to understand the stress mediated lipid accumulation in *S. quadricauda*.

As mentioned above metabolic rearrangements of cells during nitrogen starvation is induced by various stress signaling molecules. The stress signaling molecules like Reactive Oxygen species (ROS), Ca^2+^, Melatonin, Abscisic acid, etc helps the cell to sense the unfavorable environment. It eventually activates cascade of signal transductions to initiate a series of counter-reactions, which will lead to tolerance of the stressed cell. Reactive Oxygen species which are formed by redox reactions of the reactive forms of molecular Oxygen including H_2_O_2_, O_2_⋅^−^ or OH^–^ radicals during abiotic stress are recognized as signals to activate the defense response (Vranova et al., 2002) and also as a second messenger to activate several signaling cascades (Shi and Collins, 2017). The increased ROS accumulation during prolonged nitrogen starvation, leads to an oxidative damage and eventually promotes neutral lipid accumulation in *Dunaliella salina*. An increase in both ROS production and lipid peroxidation were observed under nitrogen starvation in association with increased lipid accumulation (Yilancioglu et al., 2014). Thus ROS have direct effects on neutral lipid accumulation in microalgae under nitrogen starvation. Therefore, the understanding of an initial biochemical changes under nitrogen starvation need to be unraveled.

Along with the metabolic and biochemical changes, nitrogen stress also induces alterations in an internal bimolecular pattern and morphology. As a preliminary study, the morphological variation in *S. quadricauda* was characterized in the present study. *Scenedesmus* is a pleomorphic strain which changes its morphology during nitrogen starvation as unicells or coenobia (Anusree et al., 2017). As the microalga is having these peculiar characteristics the nitrogen stress-driven morphological variation in a population of *S. quadricauda* has not been completely studied.

Finally, the stress responses may be activating the genes of lipidomic, carbon and other metabolic pathways leading to neutral lipid accumulation is remain unexplored. The major evidence for the query covers different omics approaches like transcriptomics, proteomics, lipidomics and metabolomics which explains the key regulators and proteins for TAG accumulation under nitrogen starvation (Miller et al., 2010; Boyle et al., 2012; Park et al., 2015; Javee et al., 2016). The monogenic approach may not reveal a complex reactions of stress mediated lipid accumulation. Thus the present study aims to study the initial stress associated morphological, biochemical, and metabolic changes in nitrogen stress-mediated lipid accumulation in a comprehensive manner.

## Materials and Methods

### Culturing and Induction of Nitrogen Stress

*Scenedesmus quadricauda* CASA CC202 were cultivated on Bold Basal Medium (BBM; Nichols and Bold, 1965), in a fabricated algal culture rack placed in an air conditioned Algal culture room maintained at 25°C. Philips Fluorescent tubes were used for illumination with a light intensity of 40 μmol/m^2^/s (Apogee Full spectrum Quatum meter – MQ 500) and the light–dark period was regulated by automated timer. The composition of media wherein (g/l): NaNO_3_ – 25; CaCl_2_.2H_2_O – 2.5; MgSO_4_.7H_2_O – 7.5; K_2_HPO_4_ – 7.5 KH_2_PO_4_ 17.5; NaCl – 2.5, trace elements are (mg/l) FeCl_3_.6H_2_O – 97; MnCl_2_.4H_2_O – 41; ZnCl_2_.6H_2_O – 5; CoCl_2_.6H_2_O – 2; Na_2_MoO_4_.2H_2_O – 0.75, and the media also contain vitamins (g/l) Biotin 0.1; vitamin B12 1; Thiamine 0.2. The pH of the media was adjusted to 6.8–7. The nitrogen starved medium (N^–^) completely lacks the NaNO_3_ as nitrogen source.

The nitrogen stress induction was performed in two-stage cultivation processes; in the first stage, the microalgae are grown in control (N^+^) media to obtain maximum cell density (240 × 10^6^ cells/ml). Further, in the second stage, the harvested biomass was washed twice with distilled water and re-inoculated to the nitrogen starved (N^–^) medium (Anand and Arumugam, 2015; Minhas et al., 2016; Sulochana and Arumugam, 2016).

### Effect of Nitrogen Stress Induction in the Morphology of *S. quadricauda*

#### Morphological Variation

Twenty microliter of the sample was dropped into a clean microscopic slide covered with a coverslip and the slide was allowed to stand for a few minutes. After that, the slide was observed under a light microscope (Di LEICA DM 200).

### Morphological Variation in a Population of *S. quadricauda* and Detection of Mitochondrial Membrane Potential by Flow Cytometry Analysis

The same samples were used for detecting the mitochondrial membrane potential of nitrogen stressed *S.quadricauda*. The sample of 1 ml taken from nitrogen stress-induced (N^–^) and respective control (N^+^) in the microcentrifuge tube from the three experimental replicates (*n* = 3). From that 100 μl sample was taken and diluted with 900 μl autoclaved distilled water and the pellet was obtained by centrifugation at 10,000 rpm for 10 min. Then the pellet was collected and washed twice with phosphate buffer saline (PBS) (pH 7.4). The washed pellet was fixed with 2.5% glutaraldehyde in PBS of 50 μl for 5 min. The pellet was then collected by centrifugation at 10,000 rpm for 5 min and washed with 1 ml PBS and again pelleted by centrifugation. The washed pellet was resuspended in 1 ml of Rhodamine 123 dye (SIGMA-ALDRICH, CAS No. 62669-70-9) (1 mg/ml ethanol stock) of 10 μl diluted with 990 μl of distilled water and incubated for 5 min at 20°C. After incubation, the excess dye was washed away by centrifugation and was resuspended pellet in 1 ml of PBS. Then the fluorescent intensity was analyzed by flow cytometry BD FACS Aria^TM^ II excitation at 505 nm and emission at 534 nm using software BD FACS Diva^TM^ (Morris et al., 1985; Baracca et al., 2003). The mean values of three independent replicates showing morphological variation (cell size) were plotted as a graph with standard deviation as an error bars.

### Quantification of Reactive Oxygen Species and Antioxidant Enzymes During Nitrogen Starvation

#### Measurement of H_2_O_2_

The control (N^+^) and nitrogen starved (N^–^) algal cells from three experimental replicates were harvested by centrifugation and resuspended in 0.1% w/v Trichloro Acetic Acid (TCA) solution for sonication. The total cell lysate was collected by centrifugation at 13000 rpm for 10 min. After that 0.5 ml of the supernatant was taken into fresh tubes and added 0.5 ml of 10 mM phosphate buffer (pH 7.0). To that add 1 ml of 1 M potassium iodide and mix the contents well. The absorbance of the solution was read at 390 nm. A standard curve was plotted using known concentrations of H_2_O_2_ and from that, the H_2_O_2_ concentration (μmol H_2_O_2_/gFW) of the sample was calculated (Velikova et al., 2000). The mean values of three independent of H_2_O_2_ concentration were plotted as a graph with standard deviation as an error bars.

#### Quantification of O_2_⋅^−^

The control (N^+^) and nitrogen starved (N^–^) algal cells of three experimental replicates (*n* = 3) were harvested by centrifugation, sonicated with 5 ml of 65 mM potassium phosphate buffer (pH 7.8). The cell lysate was collected by centrifugation at 12,000 rpm for 5 min. From that 1 ml of supernatant was taken into a fresh tube and mixed with 0.9 ml of 65 mM potassium phosphate buffer (pH 7.8). About 0.1 ml of 10 mM hydroxyl ammonium chloride was added to the mixture and incubated at 25°C for 20 min. After the incubation, 1 ml of 17 mM sulphanilic acid, and 1 ml of 7 mM α-naphthylamine were added to the mixture. Again the tubes were incubated for 20 min and the absorbance was read at 530 nm. The mean values of three independent replicates of O_2_⋅^−^ concentration were plotted as a graph with standard deviation as an error bars. Sodium nitrite was used to plot the standard curve from that the production of O_2_⋅^−^ was calculated (Liu et al., 2010).

#### Measurement of OH^–^

The control (N^+^) and nitrogen starved (N^–^) algal cells of three experimental replicates (*n* = 3) were harvested by centrifugation and sonicated with 2 ml of 50 mM potassium phosphate buffer (pH 7.0). Then the homogenate was centrifuged at 12,000 rpm for 5 min. From that 0.5 ml of supernatant was taken into a fresh tube and added 0.5 ml of 50 mM potassium phosphate buffer (pH 7.0) containing 2.5 mM of 2-deoxy ribose. The tubes were kept at 35°C in dark for 1 h. After the incubation 1 ml of 1% Thiobarbituric acid (TBA) in 0.5 M sodium hydroxide and 1 ml of acetic acid were added and mixed well. The tubes were boiled for 30 min and immediately cooled on ice. Further, the absorbance was read at 532 nm and the OH^–^ content was expressed as absorbance units per gram of Fresh Weight (FW; Halliwell, 2006). The mean values of three independent replicates of OH^–^ concentration were plotted as a graph with standard deviation as an error bars.

#### Lipid Peroxidation

Microalgal cells of three independent replicates from N^+^ and N^–^ samples were harvested by centrifugation at 8000 rpm for 10 min. Then the cells were sonicated in 2 ml of 80:20 (v/v) ethanol: water and the lysate were collected by centrifugation at13,000 rpm for 10 min. Further, 1 ml of the supernatant was taken into fresh test tubes and added 1 ml of TBA solution [20% (w/v) TCA, 0.01% butylated hydroxytoluene and 0.65% TBA]. The samples were mixed well and heated at 95°C for 25 min and cooled. The contents were centrifuged at 13,000 rpm for 10 min and the absorbance of the supernatant was read at 450, 532, and 660 nm (Hodges et al., 1999). The mean values of three independent replicates were plotted as a graph with standard deviation as an error bars.

Malondialdehyde (MDA) (μmol/gFW) = [6.45 × (A_532_– A_600_)] – [0.56 × A_450_]/FW

### Estimation of Antioxidant Enzymes

#### Catalase Assay

Catalase activity was determined using catalase calorimetric activity kit (Invitrogen, EIACATC). Nitrogen stressed (N^–^) and control (N^+^) algal pellet (100 mg) were collected by centrifugation at 8000 rpm for 10 min. Further, the pellet was homogenized or sonicated in 1 ml of cold 1× assay buffer (as provided by the manufactures) per 100 mg of cells. Then the content was centrifuged at 10,000 rpm for 15 min at 4°C. Collect the supernatant and assay immediately, or store at ≤−70°C.

As dilution of standards for catalase assay was prepared as described by the manufactures instructions. In brief, one unit of catalase decomposes 1 μmol of H_2_O_2_ per minute at pH 7.0 and 25°C. About 10 μl of catalase standards was added to one tube containing 190 μl 1× assay buffer and labeled as 5 U/ml catalase. 100 μl of 1× assay buffer was added to each of six tubes labeled as follows: 2.5, 1.25, 0.625, 0.313, 0.156, and 0 U/ml catalase. Serial dilutions of the standard were prepared as described in the kit manual.

Accurately 25 μl of standards or diluted samples were added to the appropriate wells. Then added 25 μl of H_2_O_2_ reagent into each well and incubated for 30 min at room temperature. After that 25 μM of the substrate was added into each well. Again added 25 μl of 1× Horse Radish Peroxidase (HRP) solution into each well and incubated for 15 min at room temperature, further, the absorbance was read at 560 nm. Curve fitting software with a four-parameter algorithm (Graph pad prism2) was used to generate the standard curve and catalase activity of samples. The mean values of three independent replicates were plotted as a graph with standard deviation as an error bars.

#### Peroxidase Assay

The peroxidase activity was quantified using Peroxidase activity assay kit (SIGMA-ALDRICH, MAK092). As dilution of standards for peroxidase assay was prepared as described by the manufactures instructions. In brief, about 10 μl of the 12.5 mM H_2_O_2_ solution was diluted with 1240 μl of assay buffer to prepare a 0.1 mM standard solution. Then 0, 10, 20, 30, 40, and 50 μl of the 0.1 mM standard solution was added into a 96 well plate, generating 0 (blank), 1, 2, 3, 4, and 5 nmol/well standards. Further, the assay buffer was added to each well to make up the volume to 50 μl. To each standard curve well, 50 μl of the standard curve reaction mix was added. Each well was mixed well and incubated at room temperature for 5 min and absorbance was read at 570 nm.

About 10 mg of the algal pellet (N^+^ and N^–^) was sonicated with 150 μl of assay buffer and centrifuged at 15,000 rpm for 10 min. Then 50 μl of the master reaction mix was added to each sample and positive control well. The contents in the well were mixed well by pipetting and incubated the plate at 37°C for 3 min, then the initial measurement was read at 570 nm (T initial). The assay was performed in the dark. The measurements were taken until the value of the test exceeded that of the standard. The final measurement [(A_570_) final] for calculating the enzyme activity would be the value before the most active sample is near or exceeds the end of the linear range of the standard curve. The time of the penultimate reading is T final.

The change in measurement from T initial to T final for samples was calculated.

$$\Delta A_{570}=\left(A_{570}\right)\text{final}-\left(A_{570}\right)\text{initial}$$

The Δmeasurement value (ΔA_570_) of each sample was compared to the standard curve to determine the amount of H_2_O_2_ reduced during the assay between T initial and T final (B). The Peroxidase activity of a sample was determined by the following equation:

Peroxidase Activity = [B × Sample Dilution Factor]/(Reaction time) × V

B, Amount (nmol) of H_2_O_2_ reduced between T initial and T final; Reaction Time, T final–T initial (min); V, Sample volume (ml) added to well.

Peroxidase activity reported as nmol/min/ml = milliunit/ml, where one unit of peroxidase is defined as the amount of enzyme that reduces 1.0 μmole of H_2_O_2_ per minute at 37°C. The mean values of three independent replicates of peroxidize activity were plotted as a graph with standard deviation as an error bars.

#### Targeted Metabolite Analysis by LC-MS

The *S. quadricauda* cells were collected by centrifugation at 8000 rpm for 10 min at room temperature. Metabolites were extracted from control or different time point samples from two independent biological replicate by homogenizing with liquid nitrogen in a prechilled sterile mortar and pestle. The samples then suspended with a mixture of 1 ml of methanol: water (80:20). Subsequently, the supernatant was collected by centrifugation at 8000 rpm for 10 minutes at 4°C and the extracted metabolites were stored at −20°C for LC-MS analysis. The mobile phase used for LC-MS is a mixture of triethylamine (A, 60%) and methanol (B, 40%) containing 0.1% formic acid adjusted to pH 4.2 and separated through a 1.9 μM C18 Shimadzu shim pack GISS column (Dimension 2.1 mm × 150 mm). The column temperature was maintained at 4°C and the temperature of the drying gas in the ionization source was 300°C. The gas flow was 10 l/min and the capillary voltage was 4 kV and the detection was using electrospray ionization (ESI)-MS. The LC-MS 8045 (Shimadzu, Japan) chromatogram was analyzed and the results were plotted by a heat map. The mean values of two experimental results were calculated and the data were used for the heat map generation (Supplementary Table 1). The heat map was generated using heat mapper (an online tool to interpret the metabolomic analysis) (Babicki et al., 2016).

#### Statistical Analysis

All the experiments were carried out in triplicate unless otherwise specified. The results are represented as mean value ± standard deviation with error bars in the figure. The data were analyzed by one-way ANOVA and the *P* value was calculated using Tukey HSD test.

## Results and Discussion

### Nitrogen Stress-Induced Morphological Variation in a Population of *S. quadricauda*

**FIGURE 1:**
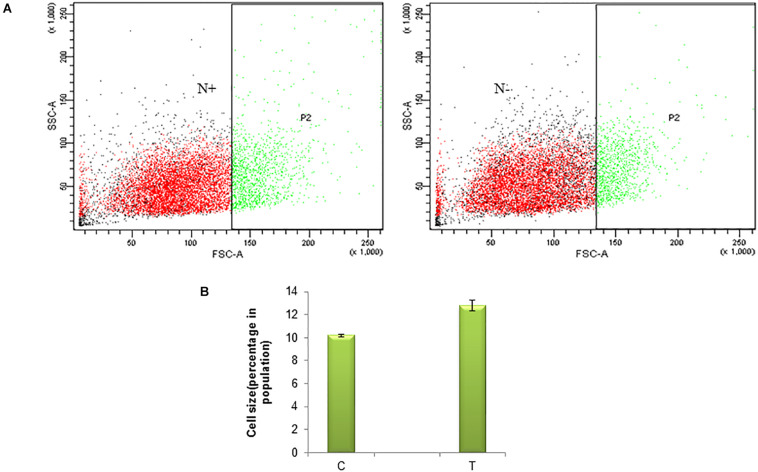
Nitrogen stress induced morphological variation in a population of *S. quadricauda.* **(A)** Flow cytometry analysis of control (N^+^) and nitrogen starved (N^–^) *S. quadricauda* P2 represents the population and SSC-A, FSC-A represents the Side Scattered light-Area. Forward Scattered light-Area respectively. **(B)** Population statistics of enlarged cells during nitrogen starvation (T) and control *S. quadricauda.* The mean values of three independent triplicates (*n* = 3) were plotted with standard deviation as error bars.

Nitrogen being an integral part of biomolecules such as proteins of an organism and thus it’s deficiency in the medium affects the enzymes required for cell division and eventually the growth of microalgae. As a primary analysis, microscopic images were observed. The nitrogen stress-induction leads to morphological changes and cell death (Supplementary Figure 1a,b). Morphological changes studied in a few cells under a microscope will not represent the phenomena at population level. Thus the variation in size of the cell due to nitrogen stress induction was studied in a population of *S. quadricauda* by flow cytometry analysis. The forward scatter analysis, the control cells were gated in such a way that large cells were presumed to represent 10% of the population and it compares to nitrogen stressed cells. The gated region represents the “region of hypertrophy” (Figure 1A). The population statistics of the cell enlargement showed that there is a 2.6% cell size enlargement in nitrogen stress-induced *S. quadricauda* (Figure 1B). The values were obtained from three independent replicates and standard deviation as an error bars.

According to Anand and Arumugam (2015), the cell size of *S. quadricauda* was enlarged in nitrogen starved condition. The accumulation of lipid droplets in *S. quadricauda* leads to variation in cell size. Similarly, the cell length was doubled in *Acutodesmus dimorphus* under nitrogen starved conditions (Chokshi et al., 2017). *Symbiodinium*, when cultured under nitrogen stress, the average cell size was observed as 7.35 and 6.96 μm at day 5 and 7 when compared to control (6.54 μm). Moreover, significant changes in the size and lipid droplets induced the morphological changes in *Scenedesmus obtusiusculus* and *Symbiodinium* during nitrogen starvation (Jiang et al., 2014).

### Biochemical Changes During Nitrogen Starvation

#### Changes in Mitochondrial Membrane Potential During Nitrogen Starvation

**FIGURE 2:**
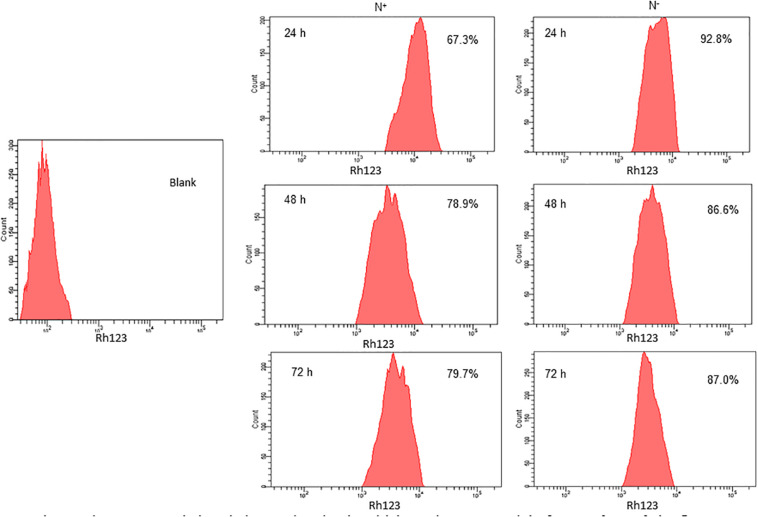
Nitrogen stress induced changes in mitochondrial membrane potential of 5. *quadricauda* by flow cytometry. Mitochondria were stained by Rhodamine123. Blank represents the auto fluorescence of *S.quadricauda*; N^+^-control. N^–^ – Nitrogen starved *S. quadricauda* at 24 h. 48 and 72 h. The experiments were carried out in duplicates (*n* = 2). The percentage values in each histogram represents the percentage cells retained the fluorescence of Rh 123 and the population of cells were fixed at 8000.

**N**itrogen stress induces perturbations in mitochondrial membrane potential (Δψ_m_), which is one of the signals to the stress through mitochondria. The increased mitochondrial membrane potential is directly proportional to the increased fluorescence of Rhodamine 123 (Rh123). The higher the Δψ_m_, the more Rhodamine 123 is taken up into the matrix. Also, the increased mitochondrial membrane potential leads to an elevated ROS generation. Here in *S. quadricauda* the rate of fluorescence of Rh 123 is increased during nitrogen stress. Eight thousand cells were taken to analyze the population and from that the fluorescence retained by the cells were represented in percentage. Thus is a 25% elevation in fluorescence of Rh 123 retained in the mitochondria of 24 h nitrogen stress-induced *S. quadricauda* when compared to control cells (Figure 2). Also, the 48 and 72 h samples revealed an increased membrane potential during the onset of nitrogen stress. Thus it implies that there is a fluctuation in mitochondrial membrane potential during onset of nitrogen stress. As the stress progresses, increased ROS generation observed in mitochondria and which eventually leads to metabolic rearrangements.

Mitochondrion plays a major role in cellular adaptation to abiotic stresses and is known to induce oxidative stress (Pastore et al., 2007). Mitochondrial membrane potential (Δψ_m_) is the driving force for ATP synthesis in mitochondria and it is generated by the proton-pumping electron transport chain. It has been reported that a correlation between membrane potential and ROS, as it generates more ROS at high membrane potential (Suski et al., 2012). Similarly, mitochondrial membrane potential and ROS generation were elevated in *S. quadricauda* during nitrogen starvation. Rhodamine 123 is a cationic, lipophilic fluorescent probe used to assay mitochondrial membrane potential in populations of apoptotic cells and it was measured according to the rate of fluorescent decay which is proportional to the mitochondrial membrane potential (Baracca et al., 2003).

### Reactive Oxygen Species Generation During Nitrogen Stress-Mediated Lipid Accumulation

**FIGURE 3:**
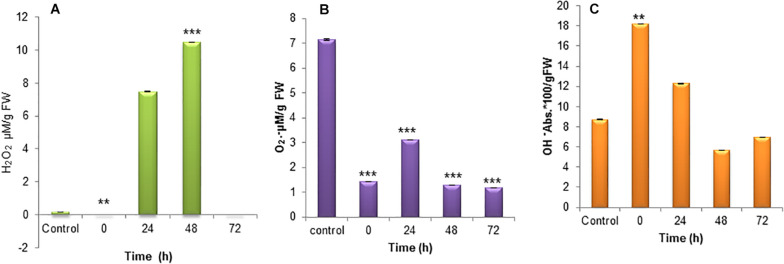
Reactive Oxygen species generation in nitrogen stress induced (N^–^) S. *quadricauda* and control (N^+^). **(A)** H_2_O_2_, **(B)** O_2_⋅^−^, and **(C)** OH^–^ generation during nitrogen stress induced at 0, 24, 48, and 72 h and the control *S. quadricauda.* The experiments were carried out in triplicate (*n* = 3) and the values were represented as a mean value with ± standard deviation as error bars. One-way ANOVA followed by Tukey HSD test for each treatment with respect to control. **Iindicate significant differences compared to control (*P* < 0.01). ***Indicates highly significant differences compared to control (*P* < 0.001).

The mitochondria are the primary producers of ROS and also it depends on the metabolic state of mitochondrion during nitrogen stress. The increased production of ROS is a sign of stress at a molecular level and the subsequent accumulation of oxidative damage. The H_2_O_2_ accumulation during nitrogen stress-induced *S. quadricauda* showed an elevated level at 24 and 48 h (*P* < 0.0001) of incubation around 7 and 11 μM respectively compared to control (0.17 μM) (Figure 3A). The O_2_⋅^−^ radical in the nitrogen stressed *S. quadricauda* showed around 3.09 μM on 24 h of incubation and it was a lower concentration compared to nitrogen-rich *S. quadricauda* where the O_2_⋅^−^ concentration was about 7.13 μM (Figure 3B). The O_2_⋅^−^ concentration in the treated samples (N^–^) found to be significant at 99% confidence level with respect to control. Also, the level of hydroxyl radical elevated during the initial hours of nitrogen stress induction (Figure 3C).

**FIGURE 4:**
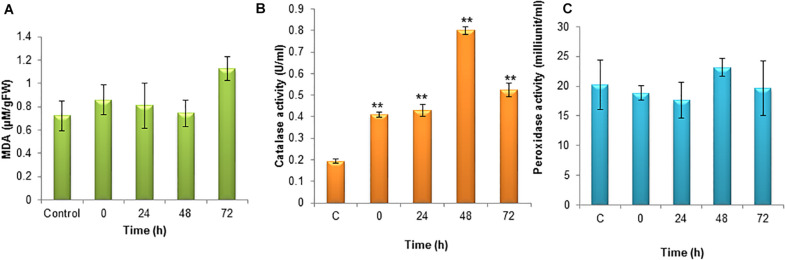
**(A)** Level of lipid peroxidation (MDA) under nitrogen stress induction in *S. quadricauda*. (B) Catalase activity in nitrogen stress induced (N^–^) *S. quadricauda* and control (N^+^). (C) Peroxidase activity in nitrogen stress induced (N^–^) *S. quadricauda* and control (N^+^). The experiments were carried out in three independent triplicates (*n* = 3) and standard deviation were represented as error bars. One-way ANOVA followed by Tukey HSD test for each treatment with respect to control. **Indicate significant differences compared to control (*P* < 0.01).

Lipid peroxidation is the oxidative degradation of lipids. The free radicals steal electrons from the membrane lipids and cause severe cell damage. Lipid peroxidation was determined in terms of MDA content in the cells. The MDA was elevated during the 72 h of incubation and it was around 1.13 μM. The MDA content was lower during the initial hours of stress induction (Figure 4A).

Even though ROS are highly reactive and potent toxic to the cells, they are having beneficial roles in abiotic stress. These include (i) diversion of electrons from the photosynthetic machinery in chloroplast to prevent the overload of the antenna and subsequent damage (Choudhury et al., 2017); (ii) regulation of metabolic fluxes during abiotic stress; and vital role as (iii) mediating signal transduction reactions which make the cells adapt to the stress by activating other pathways (Vaahtera et al., 2014; Considine et al., 2015; Mignolet-Spruyt et al., 2016; Mittler, 2017). Chokshi et al., 2017, reported that 3 days nitrogen starved *Acutodesmus dimorphus* showed 2-fold elevated levels of H_2_O_2_ than the control and simultaneously 4-fold reduction in O_2_⋅^−^ in nitrogen starved cells. H_2_O_2_ and O_2_⋅^−^ are showing the inverse relationship, as highly reactive O_2_⋅^−^ is converted into H_2_O_2_ by the enzyme superoxide dismutase (SOD). According to them the OH- and MDA did not vary significantly in nitrogen stressed *A. dimorphus*. But in *Chlorella sorokiniana* C3 showed a significant increase in the MDA level during nitrogen starvation-induced oxidative stress (Zhang et al., 2013).

### Antagonistic Antioxidant Enzymes During Nitrogen Stress

#### Catalase and Peroxidase Activity in *S.quadricauda* Under Nitrogen Stress

In order to clear-off the highly ROS, the free radical scavenging enzymes were also elevated during nitrogen stress induction. Catalase is the enzymes which speed up the conversion of H_2_O_2_ to water and oxygen. During nitrogen stress, H_2_O_2_ generation was elevated at the same time the catalase activity was also found to be increased significantly (*P* < 0.01). The catalase activity was observed to be about 0.8 U/ml (Figure 4B).

The peroxidase is heme-containing proteins which catalyze the conversion of H_2_O_2_ into the water and an activated donor molecule. It utilizes H_2_O_2_ from various organic and inorganic substrates. Relatively, peroxidase enzyme in *S. quadricauda* was less active for H_2_O_2_ oxidoreduction. As it was evidenced from Figure 4C, there is a deviation in peroxidase activity at 48 h of nitrogen stress induction (23.16 mU/ml) compared to control (20.22 mU/ml).

The overproduction of toxic ROS was neutralized by the antioxidant scavenging enzymes such as SOD, catalase and ascorbate peroxidase during nutrient starvation (Ali et al., 2005; Bhaduri and Fulekar, 2012; Fan et al., 2014; Yilancioglu et al., 2014; Ruiz-Dominguez et al., 2015; Salbitani et al., 2015). Also, the Yilancioglu et al. (2014), in *D. salina* observed an elevated level of catalase and peroxidase activity under nitrogen-deficient conditions. Their experimental evidence suggested that the lipid accumulation might be partially induced by ROS mediated oxidative stress under nitrogen starvation. In order to prove that, they induced oxidative stress by H_2_O_2_ and the results showed that increased lipid accumulation during induced oxidative stress with full strength nitrogen source in *D. salina*. In addition to that, they have claimed that oxidative stress itself can trigger lipid accumulation and suggested that the lipid accumulation was mediated by oxidative stress during nitrogen starvation.

### Metabolic Changes During Nitrogen Starvation

#### Targeted Stress Metabolite Analysis by LC-MS

**FIGURE 5:**
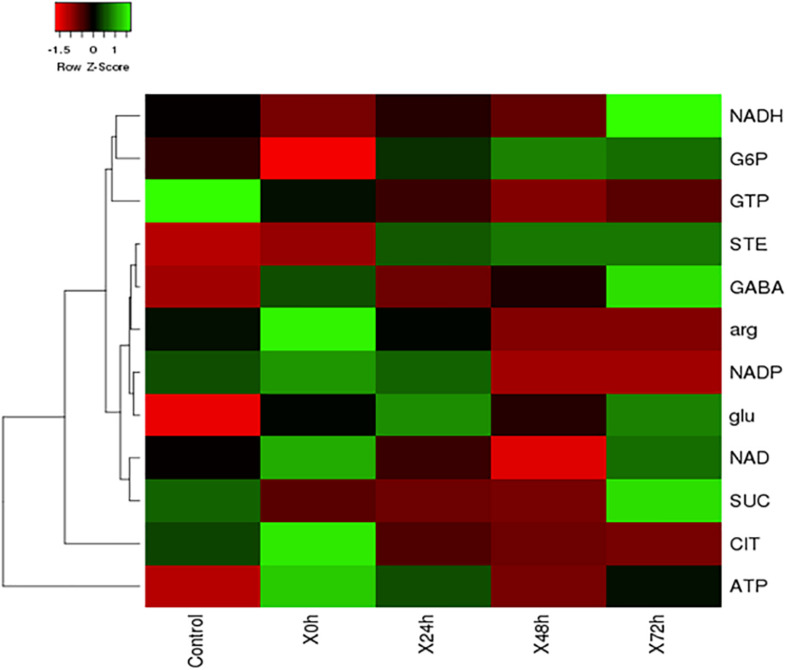
The heat map of nitrogen stress induced *S. quadricauda* at 0, 24, 48, and 72 h and the control i.e., nitrogen sufficient *S. quadricauda* represented in X axis. The targeted metabolites were represented in Y axis and the map was generated by heat mapper. The mean values of experimental duplicates (*n* = 2) were used to generate metabolite expression heat map.

During nitrogen stress, several changes are happening in the cell and the cellular events triggered by the stress, finally leads to TAG accumulation as an energy reserve. Metabolomics is one of the omics studies which help to understand the metabolic rearrangement of the cell during nitrogen stress. In order to address the metabolic changes governed by nitrogen stress, several metabolites were listed and its role was discussed in Table 1. The metabolic changes are mainly associated with the liberation of low molecular weight biomolecules and their levels during abiotic stress condition (Supplementary Table 1). The integrated targeted metabolic analysis was characterized by LC-MS analysis. The heat map results showed stress-related non-proteinogenic amino acids and energy equivalents elevated during the initial hour of nitrogen starvation (Figure 5). The non-proteinogenic amino acids like Gamma Amino Butyric Acid (GABA), glutamate and arginine were observed in maximum peak area at 72, 24 and 0 h of nitrogen stress induction respectively. Also, the energy equivalents such as NADH and ATP are highly reactive during 72, 0 h of nitrogen stress induction (Figure 5).

**TABLE 1:** Role of different metabolites induced by nitrogen stress in eukaryotes.

Targeted metabolites	Role
GABA	Regulation of energy metabolism Bypasses two steps in TCA cycle
Glutamate	Precursor of chlorophyll
Arginine	Regulation of energy metabolism
Sucrose	Promotes cell expansion and storage
Citrate	Intermediate of TCA cycle
Succinate	Intermediate between the glyoxylate cycle and TCA cycle
GTP	Regulation of energy metabolism
ATP	Regulation of energy metabolism
Glucose-6-Phosphate	Intermediate of glycolysis
NAD	Regulation of energy metabolism
NADH	Regulation of energy metabolism
NADP	Regulation of energy metabolism
NADPH	Regulation of energy metabolism

The metabolic changes during nitrogen starvation showed low molecular weight secondary metabolite accumulation and metabolic rearrangement to cope up the stress (Salama et al., 2019). To adjust the metabolic changes, microalgal species modulates their metabolite synthesis (Paliwal et al., 2017). An elevated level of sugars (glucose, sucrose, and fructose) was observed in salinity and they have a role in osmotic homeostasis, carbon storage as well as scavenging of free radicals (Rosa et al., 2009). Several researchers proposed that the fatty acid synthesis was promoted by the hyperactivity of Tricarboxylic acid (TCA) cycle (Sweetlove et al., 2010; Hockin et al., 2012; Lee et al., 2012). According to Guerra et al., 2013, the hyperactivity of TCA cycle occurs because the lipid synthesis needs more ATP together with the reduction power of NADPH during nitrogen starvation. The lipid synthesis after nitrogen starvation creates a C/N imbalance and it can be adjusted by the protein degradation to take out the amino acids. The amino acids such as leucine, isoleucine, and valine take part in the synthesis of Acetyl CoA (Allen et al., 2011; Ge et al., 2014) which is the precursor of fatty acid synthesis. Also, the glutamate forms the precursor for chlorophyll synthesis. Gamma Amino Butyric Acid is a non- protein amino acid whose levels are found to be increased during the response to nitrogen stress (Xupeng et al., 2017). The present study also indicates that the energy equivalents and non-proteinogenic amino acid-like GABA was found elevated during nitrogen starvation in *S. quadricauda*. During abiotic stress metabolites of glycolysis and TCA cycle along with these amino acids showed an initial increase in levels followed by a decrease (Zhang et al., 2016).

## Conclusion

The nitrogen stress leads to oxidative stress-induced ROS generation at high membrane potential (Δψ_m_). The predominant ROS generated were H_2_O_2_, OH^–^, O_2_⋅^−^ and in order to suppress the ROS, antioxidant scavenging enzymes like peroxidase and catalase were elevated. Also, it showed an inverse correlation between O_2_⋅^−^ and H_2_O_2_, also the OH^–^ and lipid peroxidation in terms of Malondialdehyde. The Metabolic changes are mainly associated with the liberation of low molecular weight biomolecules and their levels during abiotic stress condition. The integrated metabolic analysis revealed that stress-related non-proteinogenic amino acids and energy equivalents are elevated during nitrogen starvation.

## Data Availability Statement

The raw data supporting the conclusions of this article will be made available by the authors, without undue reservation.

## Author Contributions

MA conceptualized the study, contributed to funding acquisition, project administration, resources, supervision, writing – review and editing, conducted the experiments and analyzed the primary data, and corrected and communicated the manuscript. SS prepared the manuscript draft. Both authors contributed to the article and approved the submitted version.

## Conflict of Interest

The authors declare that the research was conducted in the absence of any commercial or financial relationships that could be construed as a potential conflict of interest.
