# Supplementary material for: Targeted Metabolomic and Biochemical Changes During Nitrogen Stress Mediated Lipid Accumulation in Scenedesmus quadricauda CASA CC202
Source: Front Bioeng Biotechnol. 2020 Oct 19;8:585632. doi: 10.3389/fbioe.2020.585632 (PMC7604524; doi:10.3389/fbioe.2020.585632)
Supplement: Supplementary file 1 [file Presentation_1.pptx]

## Slide 1
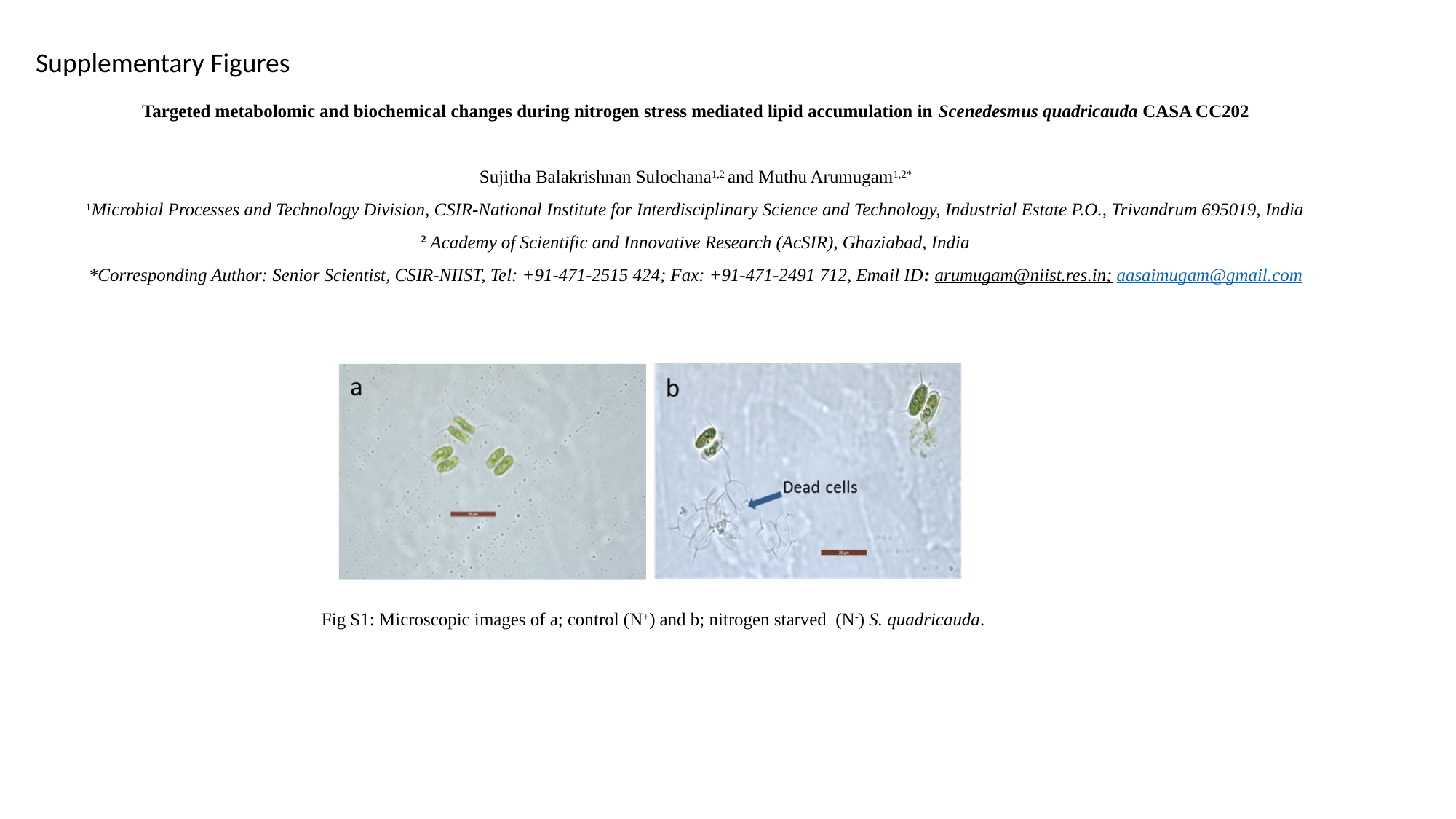

Supplementary Figures
Targeted metabolomic and biochemical changes during nitrogen stress mediated lipid accumulation in Scenedesmus quadricauda CASA CC202
Sujitha Balakrishnan Sulochana1,2 and Muthu Arumugam1,2*
1Microbial Processes and Technology Division, CSIR-National Institute for Interdisciplinary Science and Technology, Industrial Estate P.O., Trivandrum 695019, India
2 Academy of Scientific and Innovative Research (AcSIR), Ghaziabad, India
*Corresponding Author: Senior Scientist, CSIR-NIIST, Tel: +91-471-2515 424; Fax: +91-471-2491 712, Email ID: arumugam@niist.res.in; aasaimugam@gmail.com
Fig S1: Microscopic images of a; control (N+) and b; nitrogen starved (N-) S. quadricauda.
