# Supplementary material for: Targeted Metabolomic and Biochemical Changes During Nitrogen Stress Mediated Lipid Accumulation in Scenedesmus quadricauda CASA CC202
Source: Front Bioeng Biotechnol. 2020 Oct 19;8:585632. doi: 10.3389/fbioe.2020.585632 (PMC7604524; doi:10.3389/fbioe.2020.585632)
Supplement: Supplementary file 2 [file Table_1.docx]

| Metabolite | Control | 0h | 24h | 48h | 72h |
| --- | --- | --- | --- | --- | --- |
| GABA | 703968.5 | 1004063 | 776965.5 | 873432 | 1180145 |
| glu | 443661 | 574720 | 648526.5 | 551677.5 | 644556 |
| arg | 1184005 | 1896099 | 1145729 | 733938.5 | 714445.5 |
| CIT | 6532084 | 10085954 | 3498695 | 2790677 | 2571782 |
| SUC | 844696.5 | 872646.5 | 1164225 | 1208297 | 1210913 |
| GTP | 4624835 | 2570644 | 2026399 | 1414486 | 1722994 |
| ATP | 6898568 | 13299689 | 11275060 | 7838441 | 10230891 |
| SUC | 2403608 | 1486642 | 1393702 | 1333580 | 2963405 |
| G6P | 3189667 | 2872579 | 3338921 | 3469893 | 3433004 |
| NAD | 1705166 | 2241628 | 1538069 | 1001045 | 2071430 |
| NADH | 2729403 | 1462470 | 2355313 | 1735672 | 5437777 |
| NADP | 550765.5 | 731297 | 614214.5 | 10300 | 12578 |

Supplementary Table.1: The liberation of stress metabolites during nitrogen starvation in *Scenedesmus quadricauda*. The peak intensities were extracted from LC- MS chromatogram and analyzed from biological replicates. The mean values of two biological replicates were listed in the table.
